# Skeletal muscle hypertrophy: cell growth is cell growth

**DOI:** 10.1152/ajpcell.00418.2024

**Published:** 2024-07-29

**Authors:** Benjamin I. Burke, Ahmed Ismaeel, Ferdinand von Walden, Kevin A. Murach, John J. McCarthy

**Affiliations:** ^1^Department of Physiology, College of Medicine, University of Kentucky, Lexington, Kentucky, United States; ^2^Center for Muscle Biology, University of Kentucky, Lexington, Kentucky, United States; ^3^Department of Women’s and Children’s Health, Karolinska Institutet, Solna, Sweden; ^4^Department Health, Human Performance, and Recreation, University of Arkansas, Fayetteville, Arkansas, United States

**Keywords:** hypertrophy, muscle, resistance training, synergist ablation

## Abstract

Roberts et al. have provided an insightful counterpoint to our review article on the utility of the synergist ablation model. The purpose of this review is to provide some further dialogue regarding the strengths and weaknesses of the synergist ablation model. Specifically, we highlight that the robustness of the model overshadows surgical limitations. We also compare the transcriptomic responses to synergist ablation in mice and resistance exercise in humans to identify common pathways. We conclude that “cell growth is cell growth” and that the mechanisms available to cells to accumulate biomass and increase in size are similar across cell types and independent of the rate of growth.

## INTRODUCTION

We applaud Roberts et al. (1) on their insightful review response to our review article “The Utility of the Rodent Synergist Ablation Model in Identifying Molecular and Cellular Mechanisms of Skeletal Muscle Hypertrophy” (2). The authors discussed important limitations of the synergist ablation (SA) model. Although we agree with many of the points raised, the purpose of this response is to provide some further dialogue and discourse regarding some of the stated disadvantages of the rodent SA model.

## CELL GROWTH IS CELL GROWTH

In their review response, Roberts et al. (1) suggest that SA generates “an unparalleled growth stimulus unlike any other model across species.” Although we agree with the notion that SA produces supra-physiological growth in skeletal muscle, we propose the idea that many of the mechanisms available to a cell to accumulate biomass and increase in size are similar, regardless of the rate of this growth or even the cell type. For example, the target of rapamycin (TOR) system is a key regulator of cell growth conserved between yeast, *Caenorhabditis elegans,* *Drosophila*, and mammals (3). Signaling via the mammalian TOR (mTOR) has been implicated in mechanical overload (MOV)-induced skeletal muscle hypertrophy, which was first identified using the SA model, before being validated in human resistance exercise studies, as we discuss in the initial review. Beyond its role in skeletal muscle hypertrophy, mTOR signaling is implicated in robust pathological cardiac hypertrophy and cancer cell growth (4). To this end, accumulating evidence suggests that skeletal muscle hypertrophy rewires cellular metabolism to generate biomass similar to cancer cells. This metabolic reprogramming is characterized by an increase in glycolysis and its interconnected anabolic pathways (serine synthesis and pentose phosphate pathways) (5–10). Cancer metabolic reprogramming factors, including the transcription factor *MYC*, have also been implicated in skeletal muscle hypertrophy, first in response to SA (11, 12) and later in human muscle after resistance exercise (13, 14). The cancer-associated isoform 2 of pyruvate kinase (PKM2), which shifts the glucose metabolism from the respiratory chain to lactate production (aerobic glycolysis), is also increased in response to resistance exercise training in humans (15), further supporting the physiological relevance of metabolic reprogramming in human muscle hypertrophy. Overall, these findings support the concept that many fundamental mechanisms of cell growth are conserved regardless of species, cell type, rate of growth, etc., thus rendering SA as an efficacious model of studying loading-induced skeletal muscle hypertrophy. In fact, as addressed in our original article, the enhanced growth response (i.e., increased rate and magnitude of growth) induced by SA is exactly the characteristic that makes it such a useful model as a vehicle of discovery.

## THE ROBUSTNESS OF THE RESPONSE TO SYNERGIST ABLATION OVERSHADOW SURGICAL LIMITATIONS

Roberts et al. (1) raise concern with our statement that SA is a highly reproducible model. The authors accurately reference differences in surgical procedures that undoubtably result in divergent outcomes. Due to variation in surgical procedures, we join the counterpoint group in calling for rigorous control of surgical technique and subsequent reporting, especially the standardization of the sham surgery. However, this concern is present with any and all experimental techniques. There will naturally be variation from laboratory to laboratory and technician to technician. The challenge, then, is to identify models that produce outcomes that are reasonably insensitive to technical variation; SA represents one such model, consistently producing robust outcomes of muscle growth. For instance, Roberts et al. mention that some researchers favor performing less invasive surgeries to minimize fiber regeneration from the procedure. Although our laboratory does not use these exact measures, our SA surgeries do not result in appreciable fiber regeneration (minimal central nucleation or embryonic myosin heavy chain expression) (16, 17), thus demonstrating how variation in SA technique does not compromise major outcomes provided the surgeries are well controlled and executed.

The counterpoint team also mentioned the use of “dual overloads” in which both the soleus and plantaris are left intact during the SA surgery, thereby inducing MOV on both muscles. Due to our own anecdotal evidence from our laboratory across numerous technicians, we fully agree that dual overload induces severe damage to the soleus, and therefore fully concur that the efficacy of SA is limited exclusively to the plantaris muscle. It is also worth noting that some groups have studied hypertrophy in the extensor digitorum longus (EDL) muscle by excision of the tibialis anterior (TA) muscle (18). However, since the EDL is only activated during the swing phase of ambulation and undergoes exclusively concentric contractions to lift the weight of the foot during walking (19), the EDL is likely not a relevant MOV model.

## SLEDGEHAMMERS LEAVE NO STONE UNTURNED

In their review response, Roberts et al. (1) ask “if a robust (and potentially novel) molecular observation is documented with SA, must it also occur across other overload models and/or be needed for muscle hypertrophy to occur?” The authors then proceed to make an astute comparison between transcriptomic data from SA-induced hypertrophy and human resistance training. The authors posit that the drastic difference in the number of differentially expressed genes (DEGs) according to ±2.0-fold cutoffs (∼1,700 DEGs by *day 7* of SA vs. only 15 DEGs 24 h following the last bout of a 10-wk resistance exercise training program) is likely noise. Although we agree that there could be a large difference in the molecular response to rodent SA and human resistance exercise due to the “sledgehammer” effect of SA, and that not all observations present with SA are relevant to muscle hypertrophy, we would like to clarify the discussion on several points. First, if there are significantly lower DEGs in response to resistance exercise training in humans, the greater heterogeneity among the human participants is likely a major contributing factor. This heterogeneity may especially be present depending on the training status of the participants (i.e., trained vs. exercise naïve). With a greater standard deviation and variability in gene expression, test statistics (log-fold change) will tend to be lower, decreasing the power to detect true differences.

**Figure 1.**
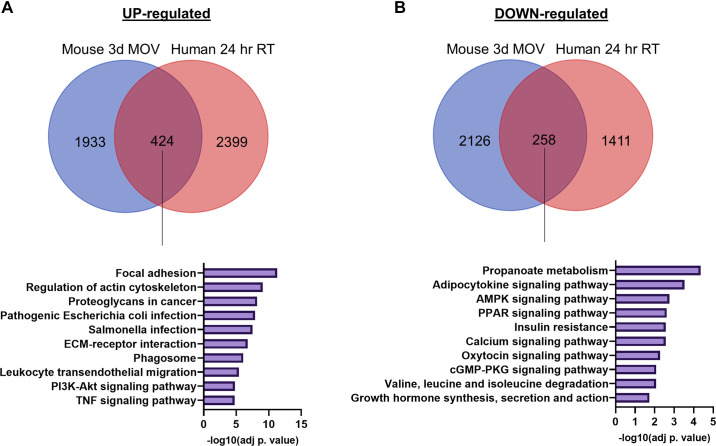
Comparison of DEGs between SA-induced MOV in mice and human resistance exercise. Venn diagrams comparing significantly upregulated genes (*A*) and significantly downregulated genes (*B*) in response to 72 h of MOV in mice and during a 24-h time course of resistance exercise in humans. The diagrams were generated using https://bioinformatics.psb.ugent.be/webtools/Venn/. Results of pathway analyses (KEGG 2021 Human) of consensus genes demonstrated in bar graphs, top 10 pathways and −log10(adjusted *P* value) shown. DEG, differentially expressed gene; MOV, mechanical overload; SA, synergist ablation; RT, resistance training.

In addition, one must keep in mind the differences in exercise mode, timing, data processing, etc., when comparing human resistance exercise with models of rodent hypertrophy. To provide a more “apples to apples” comparison, we compared RNA sequencing (RNA-Seq) data after 72 h of SA of mouse plantaris muscle (12) with RNA-Seq data from a 24-h time course of resistance exercise in human vastus lateralis muscle (13) using adjusted *P* < 0.05 for both datasets—both datasets were derived from our laboratories and processed similarly. We reported that 1,933 genes were significantly upregulated following SA in mice, and 2,399 genes were significantly upregulated followed resistance exercise in humans (Fig. 1*A*). This result is striking as more genes were upregulated with resistance exercise over 24 h in humans versus short-term SA in mice. Pathway analysis of the common upregulated genes (424) using the Enrichr tool (20–22) identified enrichment of pathways including focal adhesion, regulation of actin cytoskeleton, and proteoglycans in cancer. This analysis reinforces our abovementioned point, which is that “cancer” genes are implicated in muscle hypertrophy of mice and humans, and that “growth is growth.” In addition, 2,126 genes were significantly downregulated following SA in mice, and 1,411 genes were significantly downregulated following resistance exercise in humans (Fig. 1*B*). Pathway analysis of the consensus genes (258) between the two datasets identified enrichment of metabolic pathways [peroxisome proliferator-activated receptor (PPAR) signaling and AMP-activated protein kinase (AMPK) signaling] in the down-regulated genes. These commonalities may be related to Warburg-like metabolic reprogramming in mice and humans, which again dovetails with our abovementioned points. In addition to these data in mice and humans, Viggars et al. (23) presented a transcriptomic analysis of the response to “SpillOver” resistance training by electrical stimulation of TA muscle in rats. They identified 2,398 DEGs after 2 days of training, 2,218 DEGs after 10 days, 1,755 DEGs after 20 days, and 3,312 DEGs after 30 days of training (23). Taken together, these data further emphasize that the number of DEGs following SA is reasonable and does not necessarily reflect noise.

Moreover, Roberts et al. (1) point to “mouse bioinformatics data indicating significant alterations occur with genes associated with mitochondrial dysfunction at *days 3*, *5*, and *7*” as a potential example of enhanced noise with SA since “resistance training in humans improves aspects of mitochondrial function.” However, we argue that the downregulation of oxidative phosphorylation genes and pyruvate-stimulated mitochondrial respiration after acute MOV may be due to pyruvate shunting from the mitochondria in favor of aerobic glycolysis and biosynthetic precursor production for growth rather than dysfunctional mitochondria (24, 25). Thus, rather than this being an example of noise, the reduction in mitochondrial metabolism may be a programmed, acute response, which cannot be compared with the chronic effect of resistance exercise training.

Finally, and most importantly, a clarification is in order. When we claim that SA elevates relevant molecular and cellular events above biological “noise,” we do not mean that SA results in fewer such events so as to reveal those that are most important. On the contrary, SA produces such a robust growth stimulus that a vast array of growth-related mechanisms are implicated in the response. In other words, SA elevates individual molecular pathways that may not be detected using models providing a milder growth stimulus or may otherwise be lost in the heterogeneity of human response.

## USING SYNERGIST ABLATION TO STUDY MECHANISMS BEYOND MUSCLE HYPERTROPHY

Roberts et al. (1) make an important point that “SA likely also has physiological consequences that can extend beyond 5 days post-surgery.” In addition to the excellent examples they provide, chronic MOV induced by SA also results in an increase in Type I oxidative fibers and an increase in capillary density, which is not a major feature during human skeletal muscle hypertrophy (26). Although these mechanisms are not translatable to human resistance exercise training, we propose that the biological changes that accompany SA can still be useful to study mechanisms beyond skeletal muscle hypertrophy. For example, SA has been used as a model to investigate mechanisms of fiber-type transitions (27) and to study insulin-mediated glucose uptake (28), independent of the effects on muscle fiber hypertrophy. Therefore, although we agree that SA is best suited to study muscle hypertrophy, there are some situations where SA may provide an efficacious model for studying other biological processes.

## LIMITATIONS OF OTHER MODELS OF MUSCLE HYPERTROPHY

The counterpoint team make a compelling case for the use of other experimental models of muscle hypertrophy, both in rodents and humans. The utility of such models cannot be overstated, and there are several shortcomings of SA that are addressed by the characteristics of other models (e.g., volitional, intermittent, etc.). Many of the strengths of the SA model are noted in our original article; however, it is worth noting additional practical strengths of SA compared with other approaches. One such benefit is the condensed time frame of hypertrophy. Although other models (e.g., progressive weighted wheel running [PoWeR] and human resistance exercise) take weeks and even months to produce significant increases in muscle size, SA produces myofibrillar-mediated increases in skeletal muscle size in as little as 7 days (29). Moreover, given the potent effect of SA, smaller sample sizes are needed to detect molecular and structural alterations in response to a growth stimulus. Other models may also require singly housed animals (e.g., PoWeR) and/or great technician, time, and effort (e.g., sled pulling), whereas, overall, SA is less labor intensive with lower per diem costs.

## CONCLUSIONS

Roberts et al. (1) produced a well-written, accurate, and important article discussing the shortcomings of the SA model. We agree with the majority of their concerns and appreciate their commitment to scientific integrity and discourse. Through this response, we desire to draw attention to the purpose of the original article, that is, to highlight the SA model as a vehicle of discovery. Although the counterpoint team addressed many relevant limitations of SA, we hope the reader appreciates the utility of the SA model in producing robust and reproducible hypertrophy, which has provided the preclinical basis for better understanding the molecular and cellular mechanisms of skeletal muscle hypertrophy.

## GRANTS

This article was supported by the National Institute on Aging, National Institutes of Health; Grant/Award Number R01AG069909 (to J.J.M.) and R01AG080047 (to K.A.M.). This work was also supported by the Swedish Research Council (2022-01392) the Swedish Research Council for Sport Science (2022/10, 2023/09) to (F.v.W.).

## DISCLOSURES

No conflicts of interest, financial or otherwise, are declared by the authors.

## AUTHOR CONTRIBUTIONS

B.I.B., A.I., and J.J.M. conceived and designed research; A.I. prepared figures; B.I.B. and A.I. drafted manuscript; F.v.W., K.A.M., and J.J.M. edited and revised manuscript; B.I.B., A.I., F.v.W., K.A.M., and J.J.M. approved final version of manuscript.
